# Hashimoto Thyroiditis beyond Cytology: A Correlation between Cytological, Hormonal, Serological, and Radiological Findings

**DOI:** 10.1155/2023/5707120

**Published:** 2023-06-19

**Authors:** Sayed Ali Almahari, Reem Maki, Noor Al Teraifi, Safa Alshaikh, Nisha Chandran, Husain Taha

**Affiliations:** ^1^Department of Pathology, Salmaniya Medical Complex, Rd No. 2904, Manama, Bahrain; ^2^Department of Radiology, Salmaniya Medical Complex, Rd No. 2904, Manama, Bahrain; ^3^Department of Internal Medicine, Salmaniya Medical Complex, Rd No. 2904, Manama, Bahrain

## Abstract

**Introduction:**

Hashimoto thyroiditis is the most common cause of chronic inflammation of the thyroid gland. Ultrasound is the modality for detection, while fine needle aspiration is the gold standard method for diagnosis. Serologic markers, such as antithyroidal peroxidase antibody (TPO) and antithyroglobulin antibody (TG), are usually elevated.

**Aim:**

The main objective is to appraise the incidence of neoplasms on a background of Hashimoto thyroiditis. Our second objective is to recognize the different sonographic appearances of Hashimoto thyroiditis, to focus on its nodular and focal patterns, and to measure the sensitivity of the ACR TIRAD system (2017) when interpreted on patients with Hashimoto thyroiditis.

**Methods:**

A single-center retrospective cross-sectional study. We studied 137 cases diagnosed cytologically as Hashimoto thyroiditis from January 2013–December 2019. The data collected were analyzed using SPSS (26th edition), and ultrasounds were reviewed by a single board-certified radiologist. The ACR thyroid imaging and Data System 2017 (ACR TI-RADs 2017) and the Bethesda System for reporting thyroid cytology 2017 (BSRTC 2017) were used for reporting ultrasound and cytology, respectively.

**Results:**

The mean age was 44.66 years and the female : male was 9 : 1. Serologically, anti-Tg was high in 22 cases (38%), while anti-TPO was positive in all of the 60 cases studied. Histologically, 11 cases were diagnosed with papillary thyroid carcinoma (8%) and a single case with follicular adenoma (0.7%). Ultrasonographically, 50% of the cases showed diffuse pattern, in which 13% of them showed micronodules. 32.2% were macronodular, and 17.7% were a focal nodular pattern. 45 nodules were interpreted with the ACR TIRAD system (2017), in which 22.2% were TR2, 26.6% were TR3, 17.7% were TR4, and 33.3% were TR5.

**Conclusion:**

Hashimoto thyroiditis is a risk factor for developing thyroid neoplasms, which necessitate a proper assessment of the cytological material studied and a correlation with the clinical and radiological features. Recognizing the different types of Hashimoto thyroiditis and its variable appearances is significantly important in performing and interpreting thyroid ultrasound imaging. Microcalcification is the most sensitive parameter to discriminate between PTC and nodular type of Hashimoto thyroiditis. The TIRAD system (2017) is a useful tool for risk stratification; however, it might create unnecessary FNA studies in the setting of Hashimoto thyroiditis because of its variable appearances on ultrasound. A modified TIRAD system for patients with Hashimoto thyroiditis is important to alleviate this confusion. Finally, anti-TPO is a sensitive marker for detecting Hashimoto thyroiditis, which could be used for future referencing of newly diagnosed cases.

## 1. Introduction

Thyroid gland diseases are the most common diseases among the disorders of the endocrine system [1]. Thyroiditis is a highly prevalent pathology of the thyroid gland. It is an inflammatory process affecting the thyroid gland, whereby patients may clinically develop hyperthyroid, euthyroid, or hypothyroid status [2]. Chronic lymphocytic thyroiditis (Hashimoto thyroiditis) is the main cause of hypothyroidism among middle-aged women, but it also can affect adolescence and children [3]. It is an autoimmune disease of the thyroid gland leading to the destruction of thyrocytes and reduction of thyroid hormone levels [4]. Chronic lymphocytic thyroiditis was described for the first time by the Japanese physician Hakaru Hashimoto, hence the name [5].

Many thyroid autoantibodies can be detected in patients diagnosed with thyroid dysfunction, such as antithyroidal peroxidase antibody (TPO-Ab), antithyroglobulin antibody (Tg-Ab), and anti-TSH receptor antibody. The former two antibodies are noted mostly in patients with Hashimoto thyroiditis, while the latter antibody is detected mainly in patients with Grave's disease [6]. In some studies, anti-TSH antibodies were also detected in patients with Hashimoto thyroiditis. Their action was mainly to block the action of the TSH receptors rather than activating them [6].

Thyroid-stimulating hormone is sensitive during the detection of early hypothyroidism and marked elevation may be found in full-blown cases [7].

**Figure 1:**
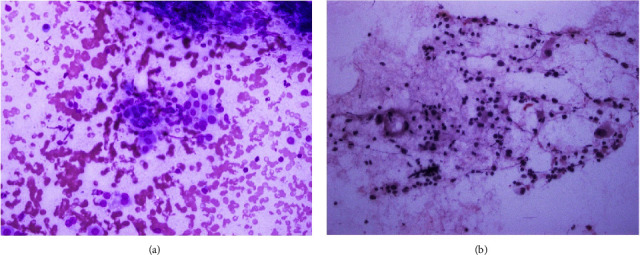
(a) Follicular cells with oncocytic change infiltrated by lymphocytic infiltrate, MGG. (b) Follicular cells with oncocytic changes infiltrated by lymphocytic infiltrate (Pap).

Cytologically, Hashimoto thyroiditis illustrates diffuse lymphoplasmacytic infiltrates, lymphoid follicles, and oncocytic cell metaplasia (Figure 1) [8].

**Figure 2:**
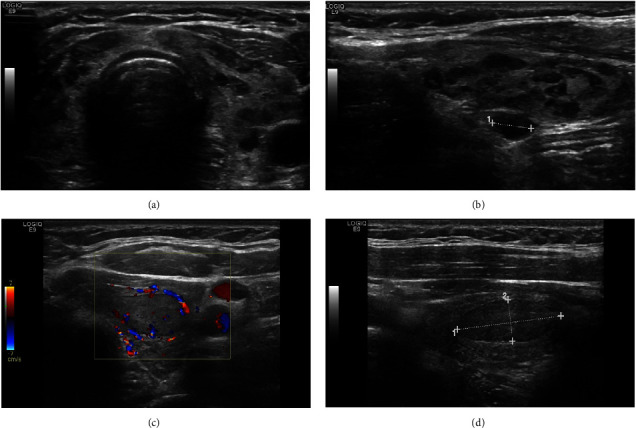
Ultrasound images for (a) and (b) diffuse lymphocytic thyroiditis with giraffe appearance. (c) and (d) Focal lymphocytic thyroiditis appears as a solid hypoechoic nodule (TR4).

Radiologically, Hashimoto thyroiditis is well recognized; however, it has variable appearances based on the disease's phase. The well-known features are mainly diffusely enlarged thyroid gland with coarsened parenchymal echotexture and hypoechoic echogenicity, and often hypervascularity on color Doppler [9, 10]. The appearance of hypoechoic micronodules with surrounding fibrous septa consistent with the micronodular pattern of Hashimoto thyroiditis is highly diagnostic and pathognomonic with a high positive predictive value [11, 12]. Discrete nodules may occur within diffusely altered parenchyma or within sonographically normal parenchyma (Figure 2) [13].

Ultrasonography is the most common modality for the detection of thyroiditis, while fine needle aspiration is considered the gold standard method for diagnosing the etiology of thyroiditis [14, 15].

In this study, we will appraise the incidence of neoplasms in patients with Hashimoto thyroiditis. Moreover, we will study the different types of Hashimoto thyroiditis and its variable appearances on ultrasonography and evaluate the sensitivity of the ACR TIRAD system (2017) on patients with Hashimoto thyroiditis. Finally, we will assess the hormonal and serological findings of patients with Hashimoto thyroiditis.

## 2. Methodology

This is a single-center retrospective cross-sectional study conducted at Salmaniya Medical Complex, Kingdom of Bahrain. The study contains 137 cases of Hashimoto thyroiditis that were diagnosed initially by fine needle aspirations (FNAs) from January 2013 to December 2019. The cases' TSH, anti-TPO, and anti-Tg results were collected along with their ultrasonographic features. The data were subtracted from the National Health Information System (I-Seha), Laboratory Information System (LIS), and Picture Archiving and Communication System (PACS).

A total number of 1222 FNAs have been performed during the study period, in which 154 FNAs were confirmed as chronic lymphocytic thyroiditis (Hashimoto thyroiditis), for the sampled 137 patients. All the patients underwent ultrasonographic-guided FNAs in the radiology department upon request from referring physicians, which was done due to three main reasons: physicians' decision, to relieve the patient's anxiety or due to imaging misinterpretation, and finally because of variable appearances of nodular and focal type of Hashimoto thyroiditis mimicking malignancies. Ultrasonography was performed using high-resolution machines with 12-5-MHz linear-array transducers (PHILIPS, FOROOGH, TOSHIBA, and SIEMENS). The scans and the procedures were performed by multiple board-certified radiologists with experience ranging between 4 and 15 years in thyroid imaging and intervention. Both longitudinal and transverse scans were obtained in grayscale. All ultrasonography images were retrospectively reviewed by one board-certified radiologist.

Our FNA method was to withdraw three to four passes using 23G needles. Moreover, a bedside adequacy check (using Diff-Quik stain) is usually performed. Half of the slides were air-dried and stained with Giemsa stain, while the other half were fixed and stained with Papanicolaou stain. A cell block is made whenever sufficient material is provided. The FNAs were reported by consultant pathologists with a minimum experience of 15–20 years in reporting thyroid cytology.

All the cases were reported using the ACR thyroid imaging and Data System 2017 (ACR TI-RADS) and the Bethesda System for reporting thyroid Cytology 2017 (BSRTC).

The patient's age, gender, operation history, type of surgery, histologic diagnosis, TSH value, anti-Tg level, anti-TPO level, and radiological features of the thyroid at the time of taking the FNA were all registered.

The age was subdivided into five groups: <20 years, 21–40, 41–60, 61–80, and more than 80.

According to our National Health Information System (i-Seha), the normal level of TSH was 0.25–0.5 mIU/L. Anti-Tg antibodies results were divided into three categories depending on the level of the antibody, either negative (<100 IU/mL), positive (>200 IU/mL), or borderline (100-200 IU/mL). For anti-TPO, the levels were divided into two categories, either negative (>28 IU/mL) or positive (>28 IU/mL).

Data were analyzed using SPSS (26th edition, IBM Corp. Software). Calculation of age means, age group frequencies, gender, operative history, surgery types, and neoplasms, along with the TSH and autoantibodies are all shown (Table 1).

Ethical approval has been obtained from the Secondary Health Care Research Committee at Salmaniya Medical Complex on 19/08/2020.

## 3. Results

A total of 137 patients underwent fine needle aspirations and were positively diagnosed with chronic lymphocytic thyroiditis (Hashimoto thyroiditis) at Salmaniya Medical Complex (January 2013–December 2019). Among which, 7 of the FNAs were repeated twice, one was repeated three times. The mean age of the patients at diagnosis was 44.66 years (ranging from 15 to 86 years). The main age group in which the patients fall into was 41–60 (69 patients). Almost 126 patients were females (91.9%), while the remaining were males (8.1%).

There were some cases in which no results were found in the system as follows: biochemistry (7 cases), anti-Tg (57 cases), anti-TPO (77 cases), and ultrasonography (13 cases). The ACR TI-RADs system was done only for 45 cases in which definite nodules were seen.

According to the Bethesda system for reporting thyroid Cytology 2017 (BSRTC) [19], 108 cases were diagnosed as Bethesda II (benign), 24 as Bethesda III (atypia of undetermined significance), 4 as Bethesda IV (follicular lesions), and 7 were Bethesda V and VI (suspicious for malignancy/malignant). Only a single case was diagnosed as Bethesda I (unsatisfactory).

Surgical interventions were performed for 42 patients (30.7%), of which 26 underwent total thyroidectomy (19%), 8 underwent right hemithyroidectomy (5.8%), 7 underwent left hemithyroidectomy (5.1%), and one underwent subtotal thyroidectomy (0.7%).

Histologically, 11 patients (8%) were diagnosed with papillary thyroid carcinoma (PTC), while one case was diagnosed with follicular neoplasm (0.7%). 44% of our positive PTC cases were below the age of 45, while the remaining (56%) above the age of 45. 

**Figure 3:**
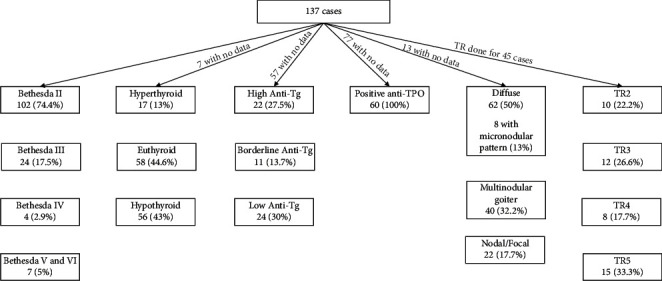
Data flow of Bethesda, TIRAD, biochemistry, and serology of the cases.

The single case of follicular neoplasm was diagnosed by cytology as Bethesda II (benign), while 4 cases of PTC were diagnosed as Bethesda V (suspicious for carcinoma), 3 as Bethesda VI (malignant), 2 as Bethesda II (benign), and 2 as Bethesda III (atypia of undetermined significance) (Figure 3).

Biochemically, most of the cases were in euthyroid status (44.6%), while 43% of the patients presented in hypothyroid status, and only 13% presented in hyperthyroid status (Figure 3).

Anti-Tg antibodies were performed for 57 patients, of which 22 were high levels, 24 were low, and 11 were borderline. On the other hand, for the anti-TPO, the levels were determined for 60 patients with positive results (Figure 3).

Radiologically, no ultrasound data were found in the Picture Archiving and Communication System (PACS) for 13 patients. Some of the FNA was done without imaging available in our system (Figure 3). Among the 124 scanned patients, the interpretation of images was based on radiologist's experience and application of the ACR TI-RADS system for detectable nodules. In our study, 62 cases showed the diffuse pattern, in which 13% of them reveal the well-known micronodular pattern with micronodules ranging from 1 to 7 mm in size. Around 1.6% of cases showed increased vascularity on color Doppler interrogation. We focused on the nodular form of Hashimoto thyroiditis and found that around 50% of the nodules were seen over a background of diffusely altered parenchyma and 40.3% of the nodules were seen over a background of normal thyroid parenchyma. We recognized that there are different clinical outcomes between nodular Hashimoto thyroiditis in a background of diffuse Hashimoto thyroiditis and nodular Hashimoto thyroiditis without it. Patients with nodular Hashimoto thyroiditis in a sonographic background of diffuse Hashimoto thyroiditis were more likely to have hypothyroidism compared to patients without it. 32.2% of the nodules were found in patients with multinodular goiter, while 17.7% were presented as focal nodules.

According to the ACR thyroid imaging and Data System 2017 (ACR TI-RADS), around 22.2% of cases were reported as benign (TR2), 26.6% were reported as mildly suspicious nodules (TR3), 17.7% were reported as moderately suspicious (TR4), and 33.3% reported as highly suspicious nodules (TR5) and pointing toward papillary thyroid carcinoma (PTC) (Figure 3).

All those nodules reported by cytology as suggestive and consistent with papillary thyroid carcinoma (PTC) were detected and interpreted as highly suspicious nodules by the ultrasound method (which account for 47% of the sonographically detected nodules). It was found that 54% of the PTCs were over a background of diffuse Hashimoto thyroiditis, while 27% were part of a macronodular pattern and 18% were focal on a background of normal thyroid parenchyma. The single case of follicular adenoma was found on a background of diffuse Hashimoto thyroiditis.

## 4. Discussion

Thyroiditis encompasses various types, including subacute, infectious, radiation-induced, palpation or trauma-induced, lymphocytic, postpartum, drug-induced, IgG4, and invasive thyroiditis [20]. Among all the types of thyroiditis, chronic lymphocytic thyroiditis (Hashimoto thyroiditis) is the most common [21].

Pathologically, it is a purely autoimmune disease caused by various types of immune cells. The thyroid gland is believed to be the essential site of thyroid antibody release, specifically from the B-cells that are heavily infiltrating the thyroid parenchyma in Hashimoto thyroiditis [22]. On the other side, the CD4 T-cells can enhance antibody production resulting in direct damage by activating CD8 cytotoxic cells and regulating the local immune reaction by the regularity of T-cells [23]. The main hypothesis for thyroid autoimmune diseases proposes having genetic susceptibility, in that, both HLA class II and non-HLA gene polymorphism have a role [24].

Most of our patients fall in the age group of (41–60 years), which goes in concordance with the other literature [4, 25, 26]. Like other studies, female predominance is observed with a male-to-female ratio of 1 : 9 [27].

There is a much-published literature comparing the levels of the anti-TPO, anti-Tg, and even some hematological parameters with the cytological grading system introduced by Chowdappa and Shetty [1, 4]. Moreover, it is documented that the higher the grade of the disease, the more progressive the disease is. Unfortunately, we have not standardized our reporting system to be used in our lymphocytic thyroiditis cases because it was not adopted by the Bethesda system for reporting thyroid cytology 2017 (BSRTC).

It is well documented that many neoplasms can be associated with Hashimoto thyroiditis, namely follicular neoplasms, oncocytic cell neoplasms, and papillary thyroid carcinoma (PTC) [28]. Also, Hashimoto thyroiditis can increase the risk of developing primary thyroid lymphomas, especially diffuse large B-cell lymphomas and MALT lymphoma [29]. The incidence of malignancy varies from 0.8 to 28% [24]. Our incidence was 8.7%, and we had mostly papillary thyroid carcinoma (PTC) and a single case of follicular neoplasm among the studied population.

For those cytologically diagnosed as Bethesda II (benign), probably it was a wrong-site aspiration, as both cases were small (microcarcinomas).

Radiologically, Hashimoto thyroiditis may present with solitary or multiple discrete nodules within either a diffusely altered parenchyma or within sonographically normal thyroid parenchyma [11, 13, 30]. This form is known as nodular Hashimoto thyroiditis, and it has an incidence of approximately 5% among nodules biopsied in previous studies, compared to 17.7% in our study [30].

The sonographic appearance of nodular Hashimoto thyroiditis is extremely variable ranging between solid, mixed solid, and cystic, hypoechoic or hyperechoic nodules. In this study, it is difficult to discriminate the true nodules from the pseudonodules found over a background of heterogeneous parenchyma and sometimes the benign nodules from the malignant ones. However, nodular Hashimoto thyroiditis shared certain features that have been previously documented pointing toward benign nodules, especially when interpreted by a trained sonographer/radiologist. These features include a sponge-like appearance, greater than 50% cystic components, hyperechoic echogenicity, and a thin surrounding regular halo [31].

On the contrary, nodular Hashimoto thyroiditis also can display certain sonographic features that are reported to be predictive of malignant thyroid nodules such as irregular hypoechoic solid nodules. This finding is troublesome and usually points toward malignancy such as papillary thyroid carcinoma (PTC), especially due to the presence of evidence suggesting Hashimoto thyroiditis as a predisposing factor for developing papillary thyroid carcinomas (PTCs) [13, 32]. Lobulated margins, a thick or irregular halo, and hypervascularity are additional features reported to be suspicious for malignancy [13, 33].

Microcalcifications are more predictable ultrasonographic features of papillary thyroid carcinoma (PTC) than of Hashimoto thyroiditis. In our study, we found a 100% accuracy result between the nodules reported as highly suspicious with high TR score due to the presence of punctate microcalcification pointing toward PTC and histology results for PTC. These results confirm those reported by others [31, 34]. Anderson et al. [34] found that all types of calcifications were more prevalent among malignant nodules within a background of diffuse Hashimoto thyroiditis. The differences were statistically significant for microcalcifications (39% vs. 0%) and for tiny nonspecific bright reflectors. More recently, Singh et al. [13] reported that none of the cases of focal Hashimoto thyroiditis had microcalcifications, whereas PTC more frequently had microcalcifications, documented in up to 70% of the considered cases. These studies' results were consistent with the results of the present study. On the contrary, 4 cases were reported as highly suspicious due to hypoechogenicity and irregular margins with no microcalcifications turned out to be histologically as Hashimoto thyroiditis. Adding to that, Sang et al. found that not only microcalcification is a predictor of PTC on a background of heterogeneous parenchyma on ultrasonography, but if aged  < 45 years, it plays an important role in predicting malignancy [35]. Despite the small sample of papillary thyroid cancer cases in our study, we found a high prevalence of around 44% in patients younger than 45 years of age, which corresponds with the aforementioned study.

We also found that the ACR thyroid imaging and Data System 2017 (ACR TI-RADS) are useful tools as a risk stratification system for malignancy in patients with Hashimoto thyroiditis. Features such as marked hypoechogenicity, taller than wider, and presence of microcalcification, are directly proportionate with high TR scoring and further evaluation with FNAC must be kept in consideration and can be used as a differentiating feature between focal Hashimoto thyroiditis and thyroid malignancy such as PTC. Smith-Bindman et al. [36] found that solid nodules significantly increase the likelihood of malignancy in the general population, whereas it was not found in Hashimoto thyroiditis patients. In our study, many benign nodules (63%) exhibited solid appearances as well as malignant ones (100%).

Despite the existence of variable TIRAD scoring systems developed to better characterize the thyroid nodules, none were tailored for the nodules that coexisted with Hashimoto thyroiditis. Kwak et al. [37] proposed to assign individual risk scores on suspicious ultrasonographic parameters to create a risk-stratifying model for thyroid nodules.

The current study encounters some limitations. First, the multiplicity of radiologists who performed the ultrasound study and the wide difference in experience became difficult to interpret the images. Second, the randomized sample of thyroid ultrasound was not dedicatedly performed for the purpose of the study. Third, due to the retrospective nature of this study, variability in US machines and operators might limit the image interpretation by reviewers. However, all the machines used in this study were high-end. According to the ACR-TIRAD system (2017), all nodules with a size less than 1 cm are not recommended for FNAC [38]; however, in our study, we received some specimens from nodules that are subcentimetric. This can lead sometimes to false-negative results, and watchful follow-up is always recommended in such scenarios.

## 5. Conclusion

To sum up, Hashimoto thyroiditis is a risk for developing thyroid neoplasms, which necessitates a proper assessment of the cytological material studied and a correlation with the clinical and radiological features.

Recognizing the different types of Hashimoto thyroiditis and its variable appearances is significantly important in performing and interpreting thyroid ultrasound imaging. Microcalcification is the most sensitive parameter to discriminate between PTC and nodular type of Hashimoto thyroiditis. The TIRAD system (2017) is a useful tool for risk stratification; however, it might create unnecessary FNA studies in the setting of Hashimoto thyroiditis due to variable appearances of the disease on ultrasound. A modified TIRAD system for patients with Hashimoto thyroiditis is important to alleviate this confusion.

Finally, we found that anti-TPO is more sensitive and specific in detecting cases of Hashimoto thyroiditis. We suggest implementing a grading system for cytology, such as the system that Bhatia et al. introduced, as it has many clinical applications, and it can suggest the severity of the disease and its complications.

## Figures and Tables

**Table 1:** Clinical and laboratory findings in cases of Hashimoto thyroiditis.

Clinical and laboratory findings	Current study	Radhi et al. [15]	Jayaram et al. [16]	Ekabaram et al. [17]	Marwaha et al. [18]
Female : male	11.4 : 1	6.14 : 1	Not recorded	Not recorded	Only young females were studied
Nodular presentation	22 (17.7%)	16 (32%)	33%	Not recorded	Not recorded
Thyroid profile	Available in 130 patients (94.8%)	Available in 41 patients (82%)	Available in 68 patients (77.27%)	Available in 50 patients (100%)	Available in all 43 patients (100%)
Hypothyroid	56 (43%)	23 (56.09%)	27 (39.7%)	42 (84%)	20%
Hyperthyroid	17 (13%)	3 (7.31%)	8 (11.7%)	3 (6%)	0%
Euthyroid	58 (44.6%)	15 (36.58%)	33 (48.5%)	5 (10%)	80%
Antibody profile	Available in 60 patients (43.8%)	Available in 11 patients (22%)	Available in 29 patients (32.95%)	Available in 40 patients (80%)	Available in 43 patients (100%)
Raised TPO-Ab	60 (100%)	9 (81.81%)	27 (93%)	26 (65%)	29 (67.4%)
Raised Tg-Ab	22 (27.5%)	7 (63.63%)	24 (82.7%)	26 (65%)	18 (41.8%)

## Data Availability

The data used to support the findings of this study were collected from the National Health Information System (I-Seha), Laboratory Information System (LIS), and Picture Archiving and Communication System (PACS).
